# Comparison of Lipid Profile in Patients With and Without Subclinical Hypothyroidism

**DOI:** 10.7759/cureus.17301

**Published:** 2021-08-19

**Authors:** Mishal Ejaz, Pardeep Kumar, Manish Murlidhar, Parkash Bachani, Sidra Naz, Kirshan Lal, Wajeeha Shahid, Simra Shahid, Maha Jahangir, Amber Rizwan

**Affiliations:** 1 Internal Medicine, Ziauddin University, Karachi, PAK; 2 Internal Medicine, Jinnah Sindh Medical University, Karachi, PAK; 3 Internal Medicine, Chandka Medical College, Larkana, PAK; 4 Internal Medicine, Liaquat University of Medical and Health Sciences, Jamshoro, PAK; 5 Internal Medicine, University of Health Sciences, Lahore, PAK; 6 Internal Medicine, Ghulam Muhammad Mahar Medical College, Sukkur, PAK; 7 Internal Medicine, Dow University of Health Sciences, Karachi, PAK; 8 Family Medicine, Jinnah Post Graduate Medical Center, Karachi, PAK

**Keywords:** lipid profile, hypothryroidism, subclinical hypothryroidism, sch, thyroxine

## Abstract

Introduction: Thyroid hormone affects lipid metabolism. Various studies have shown a contradictory relationship between lipid profile (LP) and subclinical hypothyroidism (SCH). Currently, there is a scarcity of regional data on the relationship between LP and SCH.

Methods: This longitudinal study was conducted in the internal medicine and cardiology units of a tertiary care hospital in Pakistan from September 2019 to March 2021. A total of 900 participants, of either gender and between the ages of 40 to 70 years, were enrolled in the study. Blood samples were sent to the laboratory to determine lipid and thyroid parameters. Participants were divided into two groups based on the presence of SCH.

Results: In our study, 179 (19.8%) participants had SCH. Total cholesterol (TC) and low-density lipoprotein (LDL) was significantly higher in participants with SCH compared to participants without SCH (228.41 ± 35.21 mg/dL vs. 171.21 ± 30.21 mg/dL; p-value: <0.00001) and (131.65 ± 28.22 mg/dL vs. 89.26 ± 18.52 mg/dL; p-value: <0.0001), respectively.

Conclusion: In conclusion, this study found an increased incidence of dyslipidemias in patients with SCH. It is associated with elevated TC and LDL levels, which are risk factors for cardiovascular disease and mortality.

## Introduction

In subclinical hypothyroidism (SCH), thyroid-stimulating hormone (TSH) levels are elevated in the blood, but serum free thyroxine (FT4) levels are normal. It is rather prevalent, affecting about 10% of women over the age of 55 [1]. SCH is linked to a higher risk of heart failure, coronary artery disease events, and coronary heart disease-related mortality. In addition, patients with SCH in their middle years may experience cognitive impairment, nonspecific symptoms such as weariness, and mood swings [2]. Other cardiovascular risk factors, such as blood pressure changes and increased atherosclerosis have been related to SCH. A change in lipid profile (LP) is another consequence of SCH [3]. Thyroid hormone affects lipid metabolism, and numerous studies have found that lipid levels increase as TSH levels rise [3]. Serum low-density lipoprotein-C (LDL-C) levels were observed to be higher in SCH patients in several investigations. Some of them discovered greater serum total cholesterol (TC) levels in SCH patients, whereas others discovered lower serum TC levels in SCH patients [4]. Changes in blood high-density lipoprotein-C (HDL-C) and triglyceride (TG) levels under SCH have also yielded contradictory results [5,6].

Currently, there is a scarcity of regional data on the relationship between lipid levels and SCH. To analyze and identify patients at risk of cardiovascular events, it is crucial to understand the link between lipid readings and SCH.

## Materials and methods

This longitudinal study was conducted in the tertiary care hospital's internal medicine and cardiology unit from September 2019 to March 2021. Community-dwelling participants (n = 900) of either gender, between the ages of 40 to 70 years, who were visiting the outpatient department for routine checkups, were enrolled in the study. The entire procedure was explained to the participants and their consent was taken. Participants were enrolled via consecutive convenient non-probability sampling. Ethical review board approval was taken from the institute before the start of the study. Participants with known thyroid dysfunction or participants on statin medication were excluded from the study. Furthermore, subjects on levothyroxine or antithyroid drugs, amiodarone or lithium, were also excluded from the study in addition to patients with terminal illness and patients who underwent recent surgery (within the last four weeks).

After enrollment, detailed history of the participant was taken and noted in a self-structured questionnaire. After the history, consent was taken again to withdraw 5-mL blood from the cubital vein via phlebotomy in two different vials. Blood was sent to the laboratory to determine lipid and thyroid parameters. Lipid parameters included TC, LDL-C, HDL-C, and TG levels. Thyroid parameters included TSH, FT4, and free triiodothyronine (FT3). SCH was defined as TSH levels between 5 and 10 mIU/L with normal thyroid hormones [7]. Participants were divided into two groups based on the presence or absence of SCH.

Data analysis was done using the Statistical Package for Social Sciences® software, version 23.0 (SPSS; IBM Corp., Armonk, NY, USA). Continuous variables such as age, TSH. T3, T4, low-density lipoprotein (LDL), high-density lipoprotein (HDL), TC, and TG were presented as mean and standard deviation. Categorical variables such as gender and the presence of SCH were presented by percentages and frequencies. Mean lipid values for groups with and without SCH were compared using an independent t-test. A p-value of less than 0.05 was considered statistically significant.

## Results

In our study, 179 (19.8%) participants had SCH. SCH was more common in females and participants with higher body mass index (BMI). TSH was significantly higher in patients with SCH compared to participants without SCH (6.58 ± 1.15 mIU/L vs. 3.12 ± 0.56 mIU/L; p-value: 0.0001) (Table 1).

**Table 1:** Characteristics of the participants BMI: body mass index; DM: diabetes mellitus; FT3: free triiodothyronine; FT4: free thyroxine; kg/m2: kilogram per square meter; mIU/L: milli-international units per liter; ng/dL: nanograms per decilitre; NS: nonsignificant; pg/dL: picogram/decilitre; SCH: subclinical hypothyroidism; SD: standard deviation; TSH: thyroid-stimulating hormone.

Characteristics	Participants with SCH (n = 179)	Participants without SCH (n = 721)	p-value
Age in years (Mean ± SD)	53.2 ± 14.6	52.7 ± 13.9	NS
Female	112 (62.5%)	342 (47.4%)	0.0002
Hypertension	152 (84.9%)	593 (83.3%)	NS
Smoking	56 (31.2%)	237 (32.8%)	NS
Type 2 DM	81 (45.2%)	346 (47.9%)	NS
BMI greater than 25 kg/m^2^	62 (34.6%)	166 (23.0%)	0.001
TSH (mIU/L)	6.58 ± 1.15	3.12 ± 0.56	<0.0001
FT3 (pg/dL)	342.61 ± 91.65	335.43 ± 89.21	NS
FT4 (ng/dL)	1.41 ± 0.33	1.46 ± 0.37	NS

TC and LDL were significantly higher in participants with SCH compared to participants without SCH (228.41 ± 35.21 mg/dL vs. 171.21 ± 30.21 mg/dL; p-value: <0.00001) and (131.65 ± 28.22 mg/dL vs. 89.26 ± 18.52 mg/dL; p-value: <0.0001), respectively (Table 2).

**Table 2:** Comparison of lipid parameters in participants with and without SCH LDL: low-density lipoprotein; HDL: high-density lipoprotein; mg/dL: milligram/deciliter; SCH: subclinical hypothyroidism; TC: total cholesterol; TG: triglyceride.

Lipid parameters (mg/dL)	Participants with SCH (n = 179)	Participants without SCH (n = 721)	p-value
TC	228.41 ± 35.21	171.21 ± 30.21	<0.0001
HDL	36.42 ± 10.12	37.12 ± 9.72	0.39
LDL	131.65 ± 28.22	89.26 ± 18.52	<0.0001
TG	122.61 ± 31.22	120.19 ± 31.01	0.35

## Discussion

This study compared lipid profiles among individuals with SCH and healthy controls. According to our results, individuals with SCH were more likely to be females and more likely to have a BMI of more than 25 kg/m2. It was associated with hypercholesteremia and higher LDL levels as compared to the healthy controls. There was no significant difference seen in HDL and TG levels.

Vierhapper et al. conducted a study on patients with euthyroidism, SCH, and overt hypothyroidism and observed similar LDL-C levels in euthyroid and SCH patients, and increased levels in overt hypothyroidism only [8]. Hueston et al. also stated that SCH is not associated with abnormalities in serum cholesterol or TG levels [9]. However, a study conducted in India showed similar results that the increase in LDL, TG, and TC is dependent on the increase in TSH levels. Hence, hyperlipidemia is more common in patients with SCH [10]. A cross-sectional study on 25,862 individuals in Colorado reported that changes in TSH levels were directly related to changes in lipid levels [11]. A meta-analysis done on data from 1990 to 2014 found that serum cholesterol, LDL-C, and TG were found to be higher in patients with SCH and quoted that the discrepancies in previous studies might have been a result of confounding factors [4].

The association between cholesterol levels and SCH is of great significance as dyslipidemias are associated with increased risk of hypertension, cardiovascular disease, and stroke incidence [3,12]. SCH is even considered a strong indicator of atherosclerosis and risk of myocardial infarction in elderly women [6]. Hence, lipid levels should be routinely monitored in patients with SCH to timely identify any derangements to prevent any associated adverse outcomes.

To correct the underlying cause of dyslipidemias in SCH, treatment with thyroxine may be considered; however, the beneficence of its use on LP is still a matter of debate. Several investigations carried out to observe the outcome of thyroxine supplementation in patients with SCH have reported a reduction in the levels of TC and LDL levels [13-16]. Meier et al. reported approximately 9-31% risk reduction of cardiovascular mortality in patients receiving thyroxine supplementation [13]. However, some authors have reported no significant reduction in TC and LDL levels after treatment with thyroxine [17-19]. Two of the factors that could be influencing the variance in the response of thyroxine therapy could be the baseline levels of TC and TSH levels before the start of the treatment. Patients with TSH levels >10 uU/ml or TC >240 mg/dl have shown a significant reduction in the levels of TC and LDL-C with thyroxine as compared to others [20]. However, inconsistencies in these results warrant further investigation on the use of thyroxine in patients with SCH for dyslipidemias.

To the best of our knowledge, this is the first study from this region to study the relation between lipid parameters and SCH. However, since the study was conducted in a single institute, care should be taken while inferring the result to the general population. Secondly, since it was a case-control study, a definite association could not be established. A large-scale prospective study is needed to confirm the association between dyslipidemia and SCH.

## Conclusions

In conclusion, this study found an increased incidence of dyslipidemias in patients with SCH. SCH is associated with elevated TC and LDL levels, which are risk factors for cardiovascular disease and mortality. To reduce the adverse outcomes associated with these findings, adequate monitoring of lipid profile is required along with treatment of the underlying cause in such patients. Thyroxine is one of the proposed interventions, but the evidence of its benefit is controversial.
